# Whole Transcriptome Sequencing Reveals miRNAs and ceRNA Networks in Duck Abdominal Fat Deposition

**DOI:** 10.3390/ani15040506

**Published:** 2025-02-11

**Authors:** Zhixiu Wang, Chunyan Yang, Bingqiang Dong, Anqi Chen, Qianqian Song, Hao Bai, Yong Jiang, Guobin Chang, Guohong Chen

**Affiliations:** Key Laboratory of Animal Genetics and Breeding and Molecular Design of Jiangsu Province, Yangzhou University, Yangzhou 225009, China; yangcy202109@163.com (C.Y.); bqdong2021@163.com (B.D.); 13336295886@163.com (A.C.); songqianqian8sdau@163.com (Q.S.); bhowen1027@yzu.edu.cn (H.B.); jiangyong@yzu.edu.cn (Y.J.); ghchen2019@yzu.edu.cn (G.C.)

**Keywords:** duck, ceRNA networks, abdominal fat deposition, miRNAs

## Abstract

Excessive deposition of abdominal fat will cause a waste of resources. In order to explore the key miRNAs and circRNA/lncRNA-miRNA-mRNA ceRNA regulatory network involved in regulating abdominal fat deposition, hematoxylin and eosin (H&E) staining was performed on abdominal fat tissues of ducks in the high abdominal fat rate group (HF) and low abdominal fat rate group (LF) at 21 and 42 days of age, and whole transcriptome sequencing was performed on abdominal tissues of ducks in the HF and LF groups at 42 days of age. The results showed that the number of adipocytes in ducks in the HF group was significantly higher than that in the LF group at 21 days of age (*p* < 0.001), while the number of adipocytes in ducks in the HF group at 42 days of age was significantly lower than that in the LF group (*p* < 0.001). In addition, transcriptome sequencing screened out a total of 14 differentially expressed miRNAs (10 miRNAs were significantly up-regulated, and 4 miRNAs were significantly down-regulated). By predicting the target genes of these differentially expressed miRNAs, a total of 305 target genes were obtained. Further analysis of miRNA target genes using GO and KEGG functional enrichment analyses revealed that these target genes were significantly enriched in the GnRH signaling pathway, the PPAR signaling pathway, insulin resistance, the mTOR signaling pathway, the AMPK signaling pathway, the FoxO signaling pathway, and other pathways related to adipose development. In addition, miRNA-205-x, miRNA-6529-x, miRNA-194-x, miRNA-215-x, miRNA-3074-x, miRNA-2954-x, novel-m0133-3p, and novel-m0156-5p were found to be important candidate miRNAs for abdominal fat deposition in ducks. These miRNAs were related to the expression of *FOXO3*, *LIFR*, *Pdk4*, *PPARA*, *FBN1*, *MYH10*, *Cd44*, *PRELP*, *Esrrg*, *AKT3*, and *STC2*. Based on these eight candidate miRNAs, a ceRNA regulatory network of circRNA/lncRNA-miRNA-mRNA regulating abdominal fat deposition was successfully constructed. The results of this study will provide a useful reference for accelerating the understanding of the molecular mechanism of duck abdominal fat deposition.

## Simple Summary

Fat deposition poses a huge challenge to the meat duck farming industry, as the energy required for poultry fat deposition is three times that required for lean meat deposition. However, abdominal fat is usually discarded. Therefore, excessive abdominal fat accumulation not only reduces the feed conversion efficiency of meat ducks but also leads to resource waste. In this study, the abdominal fat tissue of Cherry Valley duck × Runzhou crested white duck F2 ducks was used as the research object, and H&E staining and whole transcriptome sequencing were used to screen the key miRNAs and circRNA/lncRNA-miRNA-mRNA ceRNA regulatory networks that affect duck abdominal fat deposition. These findings deepen our understanding of the molecular mechanism of duck abdominal fat deposition and provide valuable insights for future research.

## 1. Introduction

Fat deposition is a very important problem in meat duck farming. The energy required for fat deposition in poultry is three times that of lean meat deposition. Intramuscular fat deposition can directly affect color, flavor, juiciness, tenderness, and other meat quality indicators, which are closely related to meat quality [1]. Abdominal fat is normally discarded. Therefore, excessive deposition of abdominal fat not only affects the feed conversion efficiency of meat ducks but also causes a waste of resources [2]. Therefore, how to reduce abdominal fat deposition has become a focus of attention for researchers and producers. Fat deposition in animals is caused by excessive nutritional levels and genetic basis. Genetic factors have been found to be the most important factor affecting fat accumulation [3]. Abdominal fat deposition in meat ducks is a complex process regulated by multifunctional genes and other non-coding RNAs [4,5].

More and more studies have shown that some non-coding RNAs, such as long-chain non-coding RNAs (lncRNAs), microRNAs (miRNAs), and circular RNAs (circRNAs), play important regulatory roles by forming a complex and precise post-transcriptional regulatory network [6,7]. MiRNAs play an important role in regulating gene expression, cell cycle, and organism development by inhibiting post-transcriptional gene expression by specifically binding to genes [8]. In general, one miRNA can usually regulate the expression of multiple genes, and multiple miRNAs can also jointly regulate the same target gene. A large number of miRNAs exist in adipocytes [9]. Currently, more and more studies have found that miRNAs associated with fat deposition, such as miRNA-130 [10], miRNA-27 [11], miRNA-145 [12], and miR-143 [13] regulate adipose differentiation; miR-214 [14] is involved in the regulation of glucose and lipid metabolism. It has also been found that miR-214 affects the differentiation of 3T3-L1 preadipocytes [15] and duck adipocytes [16] by targeting the expression of catenin beta 1 (*CTNNB1*) through the Wnt/β-catenin signaling pathway.

The ceRNA hypothesis was first reported several years ago, and since then, it has been widely accepted and used to understand various genetic mechanisms [17]. Zhai et al. [18]. and Xiao et al. [19] identified DElncRNAs associated with abdominal fat development and constructed a lncRNA-miRNA-mRNA interaction regulatory network. Currently, there are relatively few studies on the relationship between ceRNA regulatory networks and abdominal fat deposition.

In this study, 304 Cherry Valley ducks × Runzhou crested white duck F_2_ generation populations were used as research subjects, and the abdominal fat tissues of ducks in high and low abdominal fat rates group were sequenced in high throughput to reveal miRNAs and genes affecting abdominal fat deposition. The results of this study laid the foundation for exploring the molecular regulatory mechanism of abdominal fat deposition in ducks and provided a theoretical basis for the breeding of high-quality meat ducks.

## 2. Materials and Methods

### 2.1. Ethical Approval

All experimental birds were managed and handled following the guidelines approved by the Animal Care and Use Committee of Yangzhou University (No. YZUDWSY2017-11-07).

### 2.2. Experimental Animals and Samples

Cherry Valley duck × Runzhou crested white duck F2 ducks were provided by Shuyang Zhongke Poultry Breeding Co., Ltd., Suqian, China. All experimental ducks were raised under uniform conditions. At the age of 21 days, 20 ducks were randomly selected for body weight measurement and recording. Subsequently, these ducks underwent exsanguination and were slaughtered to extract abdominal fat tissue, which was then weighed. The abdominal fat ratio was calculated based on the proportion of abdominal fat weight to body weight. To further analyze, ducks with extreme abdominal fat ratios (i.e., the highest and lowest) were selected for histological studies. They were fasted after 42 days of feeding and weighed as live weights after 6 h of fasting, followed by slaughter and depilation. The carcasses were then dissected, and the weight of the heart, liver, gizzard, gastric antrum, abdominal fat, esophagus, trachea, crop, intestines, spleen, pancreas, gallbladder, gizzard contents, cuticle, and reproductive organs were removed as eviscerated weight (EW). The weight of abdominal fat (AF) was also recorded. The abdominal fat rate was the percentage of AF to EW. The ducks were sorted according to the size of the abdominal fat rate. Ducks with an abdominal fat rate greater than 1.75% were assigned to the high abdominal fat rate group (HF), and ducks with an abdominal fat rate less than 0.75% were assigned to the low abdominal fat rate group (LF). In order to exclude the influence of sex differences on abdominal fat deposition, male ducks were used to study abdominal fat. Four mice were selected from each of the high and low abdominal fat rate groups for whole transcriptome sequencing and were named mi-HF-1, mi-HF-2, mi-HF-3, mi-HF-4, mi-LF-1, mi-LF-2, mi-LF-3, and mi-LF-4, respectively.

### 2.3. Histological Studies

Histological examination of abdominal fat tissue was performed using hematoxylin and eosin (H&E) staining. For the histological study, one image was taken per section to ensure image representativeness and quality. Three sections were collected from each duck to obtain a sufficient sample size for analysis. A total of 12 ducks were used throughout the study to ensure data reliability and statistical power. The abdominal fat samples were washed in PBS, fixed with 4% paraformaldehyde solution, and then embedded in paraffin sections. The sections were processed by dehydration, staining (hematoxylin staining and eosin staining), dehydration, transparentization, and mounting. The samples were scanned using a Nanozoomer scanner, and the adipocyte area was calculated using an image analysis system (3DHISTECH’s Slide Converter).

### 2.4. Total RNA Extraction and Sequencing

Total RNA was extracted from abdominal adipose tissue using a Trizol kit (Invitrogen, Carlsbad, CA, USA). RNA concentration and quality were determined using a Nanodrop ND-2000 ultramicro spectrophotometer (Thermo Fisher Scientific, Waltham, MA, USA). The OD260/OD280 (Ratio, R) of the RNA was between 1.8 and 2.0, and the concentration was over 500 ng/µL. Fragments of 18-30 nt were selected using agarose gel electrophoresis cuttings. The 3′ and 5′ junctions were connected, and then reverse transcription and PCR amplification were performed on the small RNA connected to both junctions. Finally, agarose gel electrophoresis was used to recover and purify the 140 bp bands to complete the library construction. The constructed libraries were quality-controlled by Agilent 2100 and qPCR and sequenced on the machine.

### 2.5. Data Processing and Analysis

Firstly, the small RNA was obtained using preliminary filtering of the original downstream data, and then further filtering of the small RNA data was conducted. The filtering conditions were as follows: (1) filter the low-quality reads in the data to obtain high-quality reads; (2) filter out reads without 3′ junctions and intercept the sequence before the 3′ junction for later analysis; (3) filter out reads with 5′ junctions; (4) filter out reads without insertion fragments and reads with insertion fragment length less than 18 nt; (5) filter out reads containing polyA; and (6) for animal samples, filter out small RNA tags with a frequency of reads lower than 2. Finally, the small RNA clean tags sequence was obtained for subsequent analysis.

### 2.6. Standard Data Analysis

Possible rRNAs, scRNAs, snoRNAs, snRNAs, and tRNAs were found and removed from the samples as much as possible by comparing the GenBank (Release 209.0) and Rfam (11.0) databases. All of the clean tags were also aligned with the reference genome. Those mapped to exons or introns might be fragments from mRNA degradation, so these tags were removed. The tags mapped to repeat sequences were also removed. Known miRNAs were identified by comparison with known duck miRNAs in miRBase using bowtie (version 1.1.2) software. All of the unannotated tags were aligned with the reference genome. According to their genome positions and hairpin structures predicted using the software Mireap (version 2.0), the novel miRNA candidates were identified. After tags were annotated as mentioned previously, the annotation results were determined in this priority order: rRNA etc.> exist miRNA > exist miRNA edit > known miRNA > repeat > exon > novel miRNA > intron. The tags that cannot be annotated as any of the above molecules were recorded as unann.

### 2.7. miRNA Expression Profiles and Target Gene Prediction

Total miRNA consists of existing miRNA, known miRNA, and novel miRNA. Based on their expression in each sample, the miRNA expression level was calculated and normalized to transcripts per million (TPM). The edgeR software (version 4.4.2) was used for differential analysis of miRNAs. The screening criteria for differential miRNAs were Ιlog2(FC)Ι ≥ 1 and *p* ≤ 0.05. RNAhybrid (v2.1.2) + svm_light (v6.01), Miranda (v3.3a), and TargetScan (Version: 7.0) were used for target gene prediction, and then the intersection of the target gene prediction results obtained using the three methods was taken as the result of miRNA target gene prediction.

### 2.8. The ceRNA Regulatory Network

In order to explore the circRNA/lncRNA-miRNA-mRNA ceRNA regulatory networks that affect abdominal fat deposition, we performed association analysis with differentially expressed circRNAs, lncRNAs, and mRNAs obtained with RNA sequencing of the same sample (Published). Since ceRNAs are mutually regulated by miRNAs, the prediction of target genes for differential miRNAs is the first step in studying the ceRNA regulatory network. Then, the Spearman rank correlation coefficient between miRNA and candidate ceRNA was calculated for the above target gene pairs, and the target gene pairs with correlation coefficients less than or equal to −0.6 were screened to find differentially negatively correlated targeted miRNA-mRNA, miRNA-lncRNA, and miRNA-circRNA. The ceRNA theory believes that the expression levels of ceRNAs competing for the same miRNA are positively correlated, so we calculated the Pearson correlation coefficient between the expression levels of the ceRNA pairs obtained in the previous step. Here, we selected ceRNA pairs with correlation coefficients of more than 0.7 as potential ceRNA pairs. After the above screening steps, the candidate ceRNA pairs for constructing the ceRNA regulatory network were finally obtained. Cytoscape (v3.9.1) was used to obtain the ceRNA interaction relationship information to construct the ceRNA regulatory network diagram.

### 2.9. GO and KEGG Functional Enrichment Analysis

Gene Ontology (GO) is an internationally standardized classification system of gene functions, which provides a dynamically updated controlled vocabulary to comprehensively describe the properties of genes and gene products in organisms. There are three ontologies in GO, describing the molecular function, the cellular component, and the biological process in which the gene is involved. In organisms, different genes coordinate with each other to exercise their biology, and pathway-based analyses help to further understand the biological functions of genes.KEGG is the main public database on pathways.

### 2.10. Validation of Differential Genes and miRNAs

QRT-PCR was used to verify expressed gene levels. Four kinds of mRNAs and four kinds of miRNAs were randomly selected (Table 1), with GAPDH as the internal reference gene and U6 as the internal reference for miRNAs. PowerUpTM SYBRTM Green Master Mix (A25742, Thermo Fisher, Beijing, China) and LightCycler 96 Real-Time PCR Detection System (Roche, Basel, Switzerland) were used for qRT-PCR analysis of genes. The genes were reverse transcribed by FastKing one-step division of genomic first-strand cDNA (TIANGEN # KR118), and qRT-PCR analysis was performed using PowerUpTM SYBRTM green master mixture (A25742, Thermo Fisher, Beijing, China), LightCycler 96 real-time PCR detection system (Roche, Basel, Switzerland).

### 2.11. Western Blotting

Abdominal fat tissues of ducks in the high and low abdominal fat rate groups were randomly selected for protein extraction. Tissue protein lysis buffer consisted of RIPA lysis buffer (Beyotime, Beijing, China), 1% phenylmethylsulfonyl fluoride or phenylmethylsulfonyl fluoride (PMSF) (Beyotime, Beijing, China), and 1% phosphorylase inhibitor (Solarbio, Beijing, China). Protein concentration was determined using a BCA kit (Beyotime, Beijing, China). Proteins were denatured using sodium dodecyl sulfate (SDS) buffer (Beyotime, Beijing, China) and separated on 8–16% polyacrylamide gels (GenScript, Nanjing, China) at 110 V for 60 min. Proteins were then transferred (300 mA, 60 min) to polyvinylidene fluoride membranes (Bio-Rad, Hercules, CA, USA). After blocking in rapid blocking solution (NCM, Suzhou, China) for 30 min, the membranes were incubated with primary antibodies overnight at 4 °C. The samples were then incubated with diluted secondary antibodies for 2 h at room temperature. Protein bands were visualized using an enhanced chemiluminescence substrate (NCM, Suzhou, China) in a Western blot detection system (Tanon, Shanghai, China) and quantified using ImageJ software (version V1.54g). Detailed information on the antibodies is provided in Table 2.

### 2.12. Statistical Methods

The original data were imported into Microsoft Office Excel 2019 software. The quantitative expression was calculated using the 2^-ΔΔCT^ method. SPSS 22.0 software was used to perform an independent sample *t*-test to analyze the differences between the two groups, with *p* < 0.05 considered statistically significant. Graphpad Prism 9 was used for drawing.

## 3. Results

### 3.1. Histological Observation

**Figure 1:**
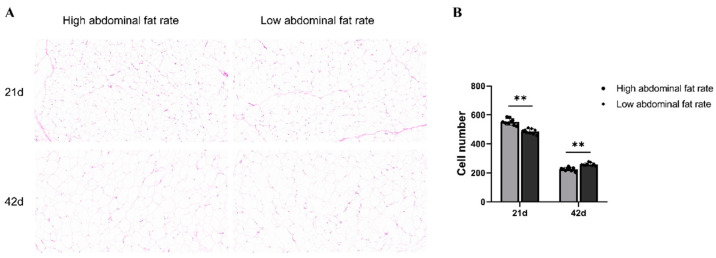
H&E staining of abdominal fat tissues of ducks in the high and low abdominal fat rate groups at 21 and 42 days of age. (**A**) H&E staining of abdominal fat tissue sections of ducks from high and low abdominal fat percentage groups under the same magnification. (**B**) Statistical histogram of the number of adipocytes in abdominal fat tissue sections of ducks under the same magnification. ** *p* < 0.001.

**Figure 2:**
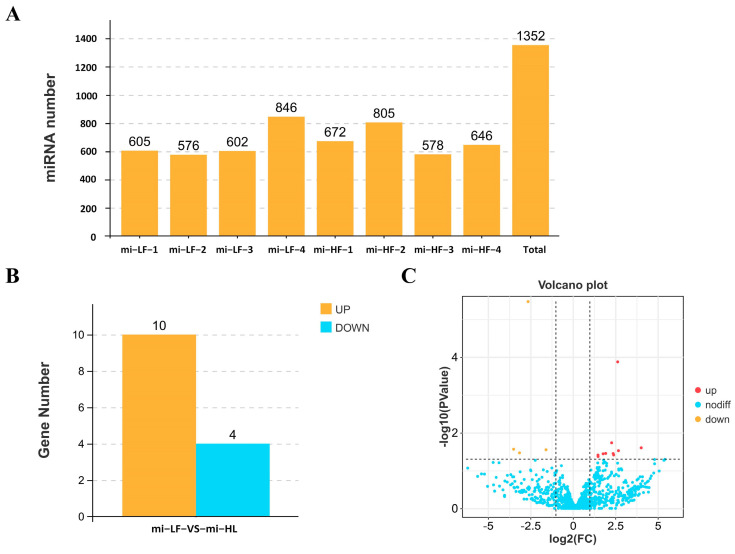
Identification and analysis of miRNA. (**A**) Statistical graph of the number of miRNAs in the sample. (**B**) Bar graph of differential miRNAs between the two groups. (**C**) Volcano graph of differential miRNAs between the two groups.

In order to better observe the differences in abdominal fat morphology between ducks in high and low abdominal fat rates groups, H&E staining was performed on the abdominal fat tissues of ducks at 21 and 42 days of age (Figure 1A). The results showed that under the same magnification, the number of adipocytes in the HF group was significantly higher than that in the LF group at 21 days of age (*p* < 0.001) (Figure 2B). The number of adipocytes in the LF group was significantly higher than that in the HF group at 42 days of age (*p* < 0.001).

### 3.2. Sequencing Data Quality Statistics

The small RNA tags obtained using preliminary screening of the original off-machine data were further quality-controlled and screened, and the results are shown in Table 3. The high-quality ratio obtained by filtering out low-quality reads in the original data in each sample was above 99%. After further filtering, the total amount of clean tags of the small RNA tags obtained was mostly above 80%. The lengths of most miRNA clean reads were 21 to 23 bp (Supplementary Figure S1), which conformed to the characteristics of miRNA and demonstrated the reliability of our data sets. The quality of sequencing data met the requirements of subsequent analysis.

### 3.3. Identification and Differential Expression Analysis of miRNA

The databases in GenBank and Rfam were selected to annotate the tag sequences obtained with sequencing, and we tried to find and remove possible rRNA, scRNA, snoRNA, snRNA, and tRNA in the sample. As shown in Supplementary Figures S2 and S3, the proportion of each sample except mi-LF-1 mapped to other non-coding RNAs (rRNA, tRNA) was less than 10%. The tag abundance of different categories in each sample is shown in Supplementary Figure S4. Finally, 1352 miRNAs were identified for further analysis (Figure 2A). Using *p* ≤ 0.05 and |log2FC| ≥ 1 as the screening criteria, a total of 14 differentially expressed miRNAs were identified, of which 10 were significantly up-regulated and 4 were significantly down-regulated (Figure 2B). The volcano plot is shown in Figure 2C.

### 3.4. Functional Enrichment Analysis of miRNA Target Genes

**Figure 3:**
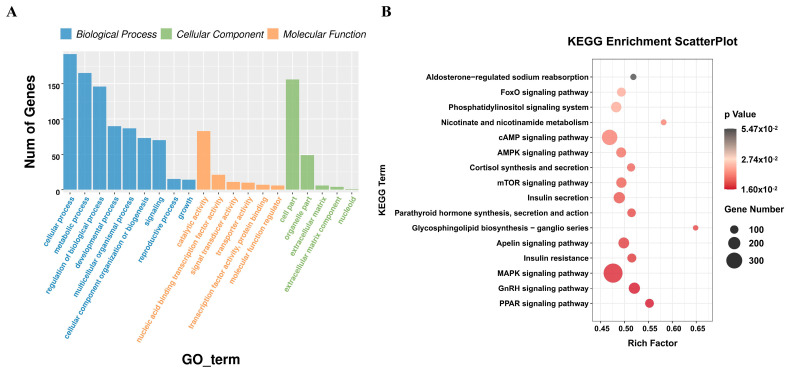
Functional enrichment analysis of miRNA target genes. (**A**) GO analysis of miRNA target genes. (**B**) KEGG functional enrichment analysis of miRNA target genes.

In order to further explore the function of miRNA, GO and KEGG functional enrichment analyses were performed on the target genes predicted by DEmiRNA. GO analysis results showed that miRNA target genes were mainly enriched in GO items such as metabolic process, nucleic acid binding transcription factor activity, regulation of biological process, and developmental process (Supplementary Table S1) (Figure 3A). KEGG results showed that miRNA target genes were significantly enriched in the GnRH signaling pathway, PPAR signaling pathway, Insulin resistance, mTOR signaling pathway, AMPK signaling pathway, FoxO signaling pathway, and other pathways related to adipose development (Supplementary Table S2) (Figure 3B). Potential gene targets involved in the regulation of adipose development were found in these adipose-related pathways, including *SOX6*, *Esrrg*, *FOXO3*, *Gli2*, *Nrf1*, *MITF*, *TBX3*, *LCAT*, *SOD3*, *EHMT1*, *Acsl4*, *AKT3*, *FKBP5*, *Traf3*, *AGTR2*, *FBN1*, *Pdk4*, *STC2*, and *GFRA1*.

### 3.5. Construction and Analysis of ceRNAs Regulatory Network

**Figure 4:**
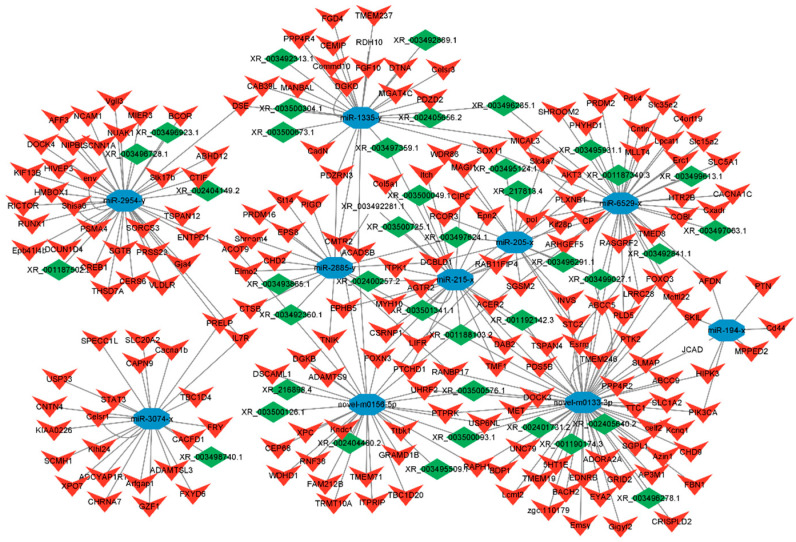
LncRNA-miRNA-mRNA CeRNA network constructed by DEmRNA, DElncRNA, and DEmiRNAs.

**Figure 5:**
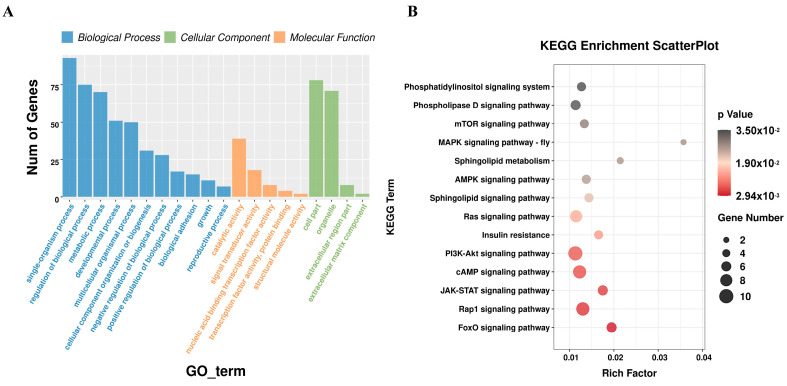
Functional enrichment analysis of mRNAs involved in the DElncRNA-DEmiRNA-DEmRNA regulatory network. (**A**) GO analysis of mRNAs involved in the DElncRNA-DEmiRNA-DEmRNA regulatory network. (**B**) KEGG functional enrichment analysis of mRNAs involved in the DElncRNA-DEmiRNA-DEmRNA regulatory network.

Based on the regulatory relationships of DEmiRNA-DEmRNA and DEmiRNA-DElncRNA, lncRNAs and mRNAs that are significantly differentially expressed and regulated by the same miRNA were screened out. Finally, 980 lncRNA-miRNA-mRNA interactions were obtained, including 60 lncRNAs, 218 mRNAs, and 10 miRNAs (Figure 4). Based on the mRNAs involved in the DElncRNA-DEmiRNA-DEmRNA regulatory network, GO and KEGG enrichment analysis was performed, and the results are shown in (Figure 5). The results showed that genes related to miRNA and lncRNA were significantly enriched in pathways related to fat development, such as the FoxO signaling pathway, mTOR signaling pathway, mTOR signaling pathway, and Sphingolipid metabolism.

**Figure 6:**
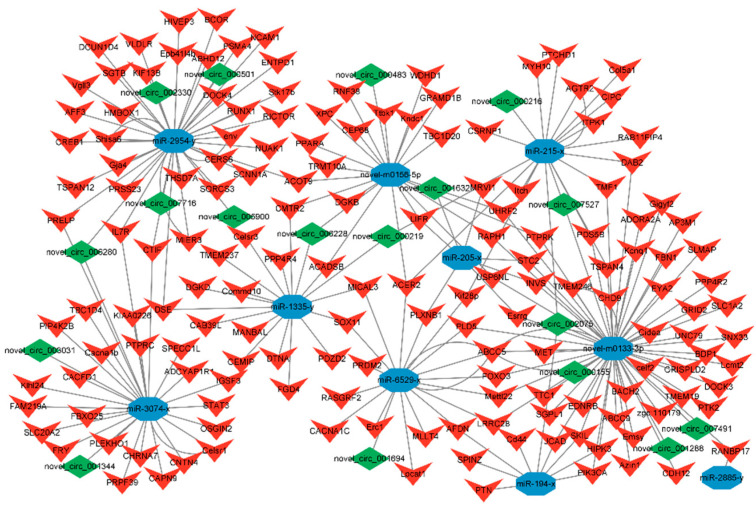
CircRNA-miRNA-mRNA CeRNA network constructed by DEmRNA, DEcircRNA, and DEmiRNAs.

**Figure 7:**
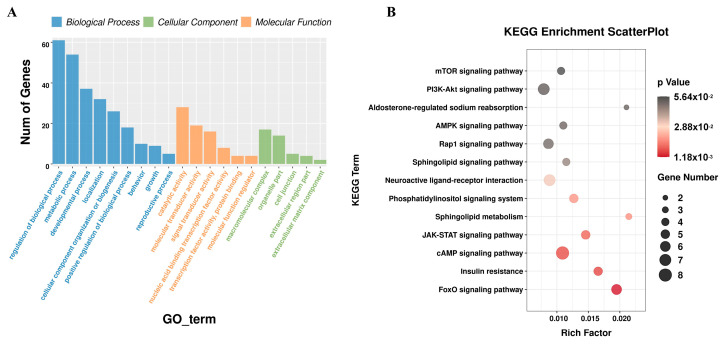
Functional enrichment analysis of mRNAs involved in the DEcircRNA-DEmiRNA-DEmRNA regulatory network. (**A**) GO analysis of mRNAs involved in the DEcircRNA-DEmiRNA-DEmRNA regulatory network. (**B**) KEGG functional enrichment analysis of mRNAs involved in the DEcircRNA-DEmiRNA-DEmRNA regulatory network.

Based on the regulatory relationships of DEmiRNA-DEmRNA and DEmiRNA-DEcircRNA, circRNAs and mRNAs that are significantly differentially expressed and regulated by the same miRNA were screened out. Finally, 370 circRNA-miRNA-mRNA interactions were obtained, including 18 circRNAs, 177 mRNAs, and 9 miRNAs (Figure 6). Based on the mRNAs participating in the DEcircRNA-DEmiRNA-DEmRNA regulatory network, GO and KEGG enrichment analysis was performed, and the results are shown in (Figure 7). The results showed that genes related to miRNA and circRNA were significantly enriched in pathways related to fat development, such as the FoxO signaling pathway, Insulin resistance, AMPK signaling pathway, mTOR signaling pathway, Phosphatidylinositol signaling system, and Sphingolipid metabolism.

### 3.6. Construction of Candidate ceRNA Network

**Figure 8:**
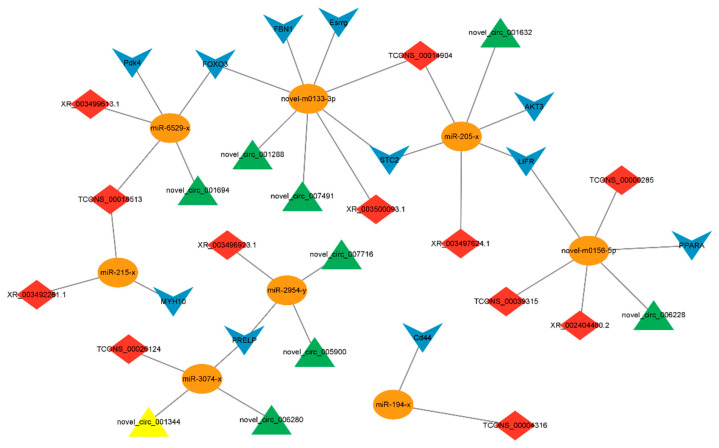
Candidate ceRNA regulatory network associated with fat deposition. Yellow circles, blue arrows, red octagons, and green triangle nodes represent co-differentially expressed miRNAs, mRNAs, lncRNAs, and circRNAs, respectively.

The study further determined the interaction relationship between candidate ceRNA transcripts. Through further screening of mRNAs related to fat development in the DElncRNA/circRNA-DEmiRNA-DEmRNA regulatory network, a total of *FOXO3*, *LIFR*, *Pdk4*, *PPARA*, *FBN1*, *MYH10*, *Cd44*, *PRELP*, *Esrrg*, *AKT3,* and *STC2* were screened to be potentially involved in regulation (Figure 8). Combined with the DEmiRNA and DEcircRNA that we screened previously that can participate in the regulation of abdominal fat deposition, a ceRNA network model involved in the regulation of fat development was established, including 8 miRNAs, 12 lncRNAs, 9 circRNAs, and 11 mRNAs.

### 3.7. QRT-PCR and Western Blot Validation Analysis

**Figure 9:**
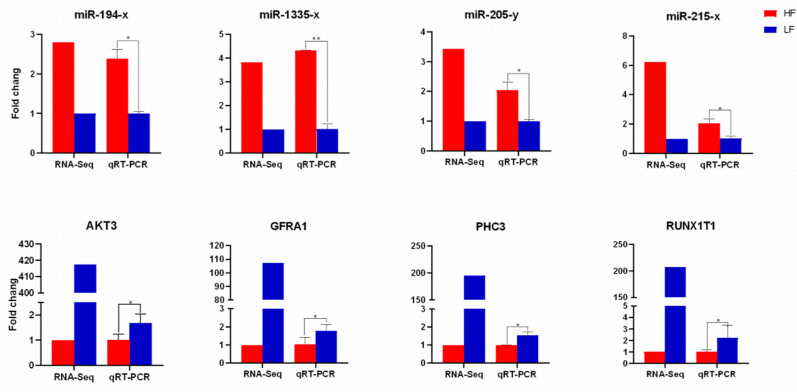
qRT-PCR verification of differentially expressed genes/miRNA results. Fold change is the fold change of FPKM in RNA-seq or fold change of relative expression in qRT-RCR. * *p* < 0.05; ** *p* < 0.01.

**Figure 10:**
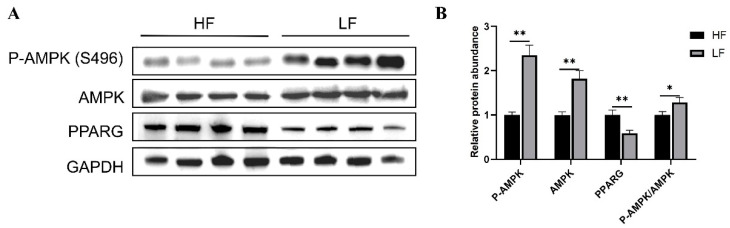
Western blot validation of AMPK signaling pathway. (**A**) Western blotting was used to detect P-AMPK, AMPK, and PPARG protein levels. (**B**) Western blotting grey value statistics. Data are means ± SEM; n = 6. * *p* < 0.05; ** *p* < 0.01.

Four differentially expressed genes and four differentially expressed miRNAs were randomly screened for qRT-PCR to verify the RNA-Seq sequencing results of the high and low abdominal fat rate groups (Figure 9). The results showed that the expression trends of the four differentially expressed genes and four differentially expressed miRNAs in high and low abdominal fat rate groups were basically consistent with the RNA-Seq sequencing results, indicating that the transcriptome sequencing data are reliable. In order to verify the enriched AMPK signaling pathway of the target gene, WB experiments were performed to detect the expression levels of AMPK, P-AMPK, and the PPARG protein in the enriched AMPK signaling pathway (Figure 10). The results showed that the P-AMPK, AMPK, and P-AMPK/AMPK ratio of low abdominal fat rate were significantly higher than those of the high abdominal fat rate group, while the protein expression of PPARG enriched in AMPK was significantly lower than that of the high abdominal fat rate group.

## 4. Discussion

In poultry production, abdominal fat is usually eliminated as a byproduct. Excessive abdominal fat deposition increases feed costs and affects duck production and quality. Therefore, exploring the key miRNAs and circRNA/lncRNA-miRNA-mRNA ceRNA regulatory networks involved in regulating abdominal fat deposition is of great significance for understanding the molecular mechanism of abdominal fat deposition in ducks. By performing H&E staining on the abdominal fat tissue of ducks at 21 days old and 42 days old, it can be seen that the difference in abdominal fat tissue between ducks in HF and LF groups at 21 days old is mainly reflected in the number of adipocytes. Normally, The number of adipocytes is fixed during embryonic and early growth and development, and the deposition of abdominal fat in later stages of growth and development is largely dependent on the ability of adipocytes to differentiate [20]. At 21 days of age, the high abdominal fat group had a large number of abdominal adipocytes, which may indicate that at this stage of growth, the ability of preadipocytes of ducks in the high abdominal fat rates group had a stronger ability to proliferate and differentiate into mature adipocytes. At 42 days of age, the number of abdominal adipocytes in the high abdominal fat rates group decreased, which may mean that these adipocytes have undergone hypertrophy, that is, the volume of individual adipocytes increases rather than the number increases, which is related to the way adipose tissue increases, including hypertrophy and hyperplasia.

Adipose tissue is the main site of energy storage and plays a role in metabolic regulation by releasing adipokines. Researchers have shown that miRNAs have important functions in fat metabolism [21,22]. For example, miRNA-18b-3p [23], miRNA-223 [24], miRNA-15a [25], and miR-21 [26] regulate adipocyte differentiation in chickens by binding to the 3′ untranslated region (UTR) of target mRNAs. miR-214 is involved in the differentiation of duck adipocytes. Studies have shown that miR-205 promotes the differentiation of 3T3-L1 preadipocytes by targeting glycogen synthase kinase 3β [27]. Elisabeth et al. [28] also found that miR-205, as a PPARγ target, is involved in the differentiation of 3T3-L1 adipocytes. This supports the results of this study. We found that miRNA-205-x was significantly highly expressed in the high abdominal fat rate group, indicating that miRNA-205-x may regulate abdominal fat deposition. Meanwhile, the target genes of miRNA-205-x, *Esrrg*, *STC2*, and *AKT3* have been associated with adipogenesis. For example, *Esrrg* regulates oxidative metabolism and mitochondrial function in adipose tissue and inhibits adipocyte differentiation when inhibited [29]. *STC2* is a secreted glycoprotein that participates in a variety of biological processes [30,31]. Related studies have shown that *STC2* is involved in glucose homeostasis and phosphorus metabolism [32,33]. Cao et al. [34] found that *STC2* may play a key role in liver lipid metabolism and muscle lipid deposition in chickens. Akt regulates numerous cellular processes, including cell growth, differentiation, proliferation, apoptosis, and glucose metabolism [35]. All three isoforms of Akt are expressed in adipocytes [36]. Ding et al. [37] found that AKT3 inhibits adipogenesis through WNK1/SGK1 signaling. This study also found that AKT3 was significantly expressed in the low abdominal fat group, indicating that AKT3 may inhibit abdominal fat deposition. The target gene is also involved in the regulation of fat, further proving that miRNA-205-x may be involved in regulating abdominal fat deposition. In addition, we also identified miRNA-6529-x, miRNA-194-x, miRNA-215-x, miRNA-3074-x, miRNA-2954-x, novel-m0133-3p, and novel-m0156-5p as potentially associated with abdominal fat deposition.

Accumulating evidence indicates that the lncRNAs and circRNAs, two classes of new ncRNAs, act as ceRNAs (or named ‘miRNA sponges’, ‘target mimics’) to function in a variety of biological processes. Based on DEmiRNAs, DElncRNAs, DEcircRNAs, and DEmRNAs, the circRNA-miRNA-mRNA and lncRNA-miRNA-mRNA regulatory networks of abdominal adipose tissue were constructed. *FOXO3*, *LIFR*, *Pdk4*, *PPARA*, *FBN1*, *MYH10*, *Cd44*, *PRELP*, *Esrrg*, *AKT3*, *RUNX1T1,* and *STC2* were found in the regulatory network of ceRNA. Studies have found that human obesity is associated with polymorphisms of the *FOXO3* gene [38]. Wan et al. [39] showed that FOXO3 regulates lipid deposition through fatty acid synthesis. *LIFR* promotes the breakdown and browning of adipocytes, leading to fat expansion and hepatic triglyceride accumulation [40]. Li et al. [41] found that knocking down *LIFR* impaired adipogenic differentiation and inhibited the expression of adipogenic marker genes *PPARγ*, *C/EBPα*, and *aP2*. *Pdk4* plays an important role in lipid-related metabolic adaptations in various tissues [42]. Pettersen et al. [43] demonstrated that *Pdk4* is closely related to fatty acid oxidation and can even serve as a sensitive marker of metabolic adaptation with an increased mitochondrial fatty acid oxidation rate. *PPARA* is a central regulator of environmentally dependent changes in fatty acid oxidation [44] and plays an important role in the regulation of lipid homeostasis and oxidative metabolism [45]. Fu et al. [46] found that *PPARA* may inhibit IMF deposition by promoting fatty acid oxidation. Zhou et al. [47] showed that *PPARA* can promote the deposition of visceral fat in chickens. *FBN1* mutations can lead to abnormalities in adipocytes [48,49]. Davis et al. [50] found that *FBN1* levels are correlated with the number of cells in adipose tissue. *FBN1* can affect the transformation from stem cells to preadipocytes and then to adipocytes. Studies have reported that *MYH10* is expressed in adipocytes. Kislev et al. [51] demonstrated that *MYH10* controls adipocyte function and adipogenesis through interaction with GLUT4. Studies have shown that *CD44* is associated with adipogenesis [52]. Weng et al. [53] demonstrated that *CD44* promotes adipogenesis by regulating PPARγ and cell cycle-related pathways. Ding et al. [54] first demonstrated that *PRELP* binds to the adipocyte membrane receptor p75NTR to affect adipocyte differentiation by activating FAK/MAPK signaling. *RUNX1T1* is a member of the runt-related transcription factor (RUNX) family of transcription factors and is associated with the number of adipocytes [55]. Deng et al. [56] found for the first time that *RUNX1T1* is an important functional molecule in adipogenesis and is negatively correlated with sheep preadipocyte differentiation. In this study, it was also found that *RUNX1T1* was significantly expressed in the low abdominal fat group, indicating that *RUNX1T1* may inhibit abdominal fat deposition. Therefore, we speculated that the above genes were involved in regulating abdominal fat deposition, and these genes were regulated by miRNA-205-x, miRNA-6529-x, miRNA-194-x, miRNA-215-x, miRNA-3074-x, miRNA-2954-x, novel-m0133-3p and novel-m0156-5p in ceRNAs related to fat deposition. These miRNAs can be used as candidate miRNAs for regulating abdominal fat deposition. Finally, with the above 8 candidate miRNAs as the core, a ceRNA network model including 8 miRNAs, 12 lncRNAs, 9 circRNAs, and 11 mRNAs was established. This ceRNA network can be used as a candidate regulatory network in duck abdominal fat deposition.

## 5. Conclusions

This study found that miRNA-205-x, miRNA-6529-x, miRNA-194-x, miRNA-215-x, miRNA-3074-x, miRNA-2954-x, novel-m0133-3p, and novel-m0156-5p can be used as important candidate miRNAs for duck abdominal fat deposition. These miRNAs target the expression of *FOXO3*, *LIFR*, *Pdk4*, *PPARA*, *FBN1*, *MYH10*, *Cd44*, *PRELP*, *Esrrg*, *AKT3,* and *STC2*. A ceRNA regulatory network of circRNA/lncRNA-miRNA-mRNA was constructed with these eight candidate miRNAs as the core. The results of this study will provide a useful reference for accelerating the understanding of the molecular mechanism of duck abdominal fat deposition.

## Figures and Tables

**Table 1:** Primer information.

Genes/miRNAs	GenBank	Primer Sequences (5′ → 3′)	Product Length/bp	Annealing Temperature/°C
miR-194-5p	-	Reverse: GTCGTATCCAGTGCAGGGTCCG AGGTATTCGCACTGGATACGACTCCCTG F: TATTGCAAACTCCGTGGCCT R: AGTGCAGGGTCCGAGGTATT	22	60
miR-215-5p	-	Reverse: GTCGTATCCAGTGCAGGGTCCG AGGTATTCGCACTGGATACGACAGTCTG F: GCGCGATGACCTATGAATTGA R: AGTGCAGGGTCCGAGGTATT	21	60
miR-205-5p	-	Reverse: GTCGTATCCAGTGCAGGGTCCG AGGTATTCGCACTGGATACGACCAGATT F: GCCCTTCATTCCACCGG R: AGTGCAGGGTCCGAGGTATT	22	60
miR-28-3p	-	Reverse: GTCGTATCCAGTGCAGGGTCCG AGGTATTCGCACTGGATACGACTCCAGG F: CGCGCACTAGATTGTGAGCT R: AGTGCAGGGTCCGAGGTATT	22	60
*AKT3*	XM_027453846	F: GGTGACGTCGGGAGTTTTC R: CTGATGAGTCACCCCCGGA	101	60
*GFRA1*	XM_021274105	F: ATTCTTCCCACATCCTGGCATT R: TCAAGCAAGTCATTTCCCTGT	149	60
*PHC3*	XM_027464250	F: CAACTGTACCACAGGCAAGC R: CTGTGCAGGTGTAGGAGAGG	125	60
*RUNX1T1*	XM_027452458	F: CATTCCAGAACAGGAGGCAC R: CTTCTCAGTGCGATCAGGCAT	105	60

**Table 2:** Details of antibodies.

Antibodies	Cat No.	Source	Predicted Band Size	Dilution of WB
P-AMPK	ET1612-72	HUABIO, Hangzhou, China	64 kDa	1:1000
AMPK	5831S	CST, Danvers, MA, USA	62 kDa	1:1000
PPARG	ab178860	abcam, Cambridge, UK	58 kDa	1:1000
GAPDH	ab181602	abcam, Cambridge, UK	36 kDa	1:5000

**Table 3:** Statistics of data filtering and junction removal for each sample.

id	clean_reads	high_quality	3′adapter_null	smaller_than_18nt	polyA	low cutoff	clean_tags
mi-HF-1	16836125	99.32%	0.38%	12.67%	0.0005%	3.19%	81.91%
mi-HF-2	30279412	99.39%	0.33%	11.42%	0.0008%	3.68%	83.57%
mi-HF-3	14406324	99.60%	0.20%	8.64%	0.0008%	4.89%	84.61%
mi-HF-4	14835442	99.40%	0.36%	13.57%	0.0006%	5.55%	79.59%
mi-LF-1	12356483	99.73%	0.15%	18.60%	0.0007%	8.22%	71.98%
mi-LF-2	15701630	99.22%	0.28%	2.27%	0.0007%	1.99%	94.95%
mi-LF-3	15106435	99.41%	0.17%	4.88%	0.0009%	3.93%	90.44%
mi-LF-4	54771598	99.26%	0.15%	2.50%	0.0005%	1.54%	95.31%

## Data Availability

Data is contained within the article or supplementary material.

## Supplementary Materials

The following supporting information can be downloaded at: https://www.mdpi.com/article/10.3390/ani15040506/s1. Supplementary Figure S1: Pie chart of tag length distribution of samples; Supplementary Figure S2: Pie chart of tag abundance of samples compared to GenBank; Supplementary Figure S3: Pie chart of tag abundance of samples compared to Rfam; Supplementary Figure S4: Pie chart of tag abundance of different classifications of samples; Supplementary Table S1: Statistical table of differential GO analysis of differential miRNA target genes; Supplementary Table S2: Statistical table of differential KEGG analysis of differential miRNA target genes.
