# Supplementary figures and images for: Whole Transcriptome Sequencing Reveals miRNAs and ceRNA Networks in Duck Abdominal Fat Deposition
Source: Animals (Basel). 2025 Feb 11;15(4):506. doi: 10.3390/ani15040506 (PMC11852153; doi:10.3390/ani15040506)

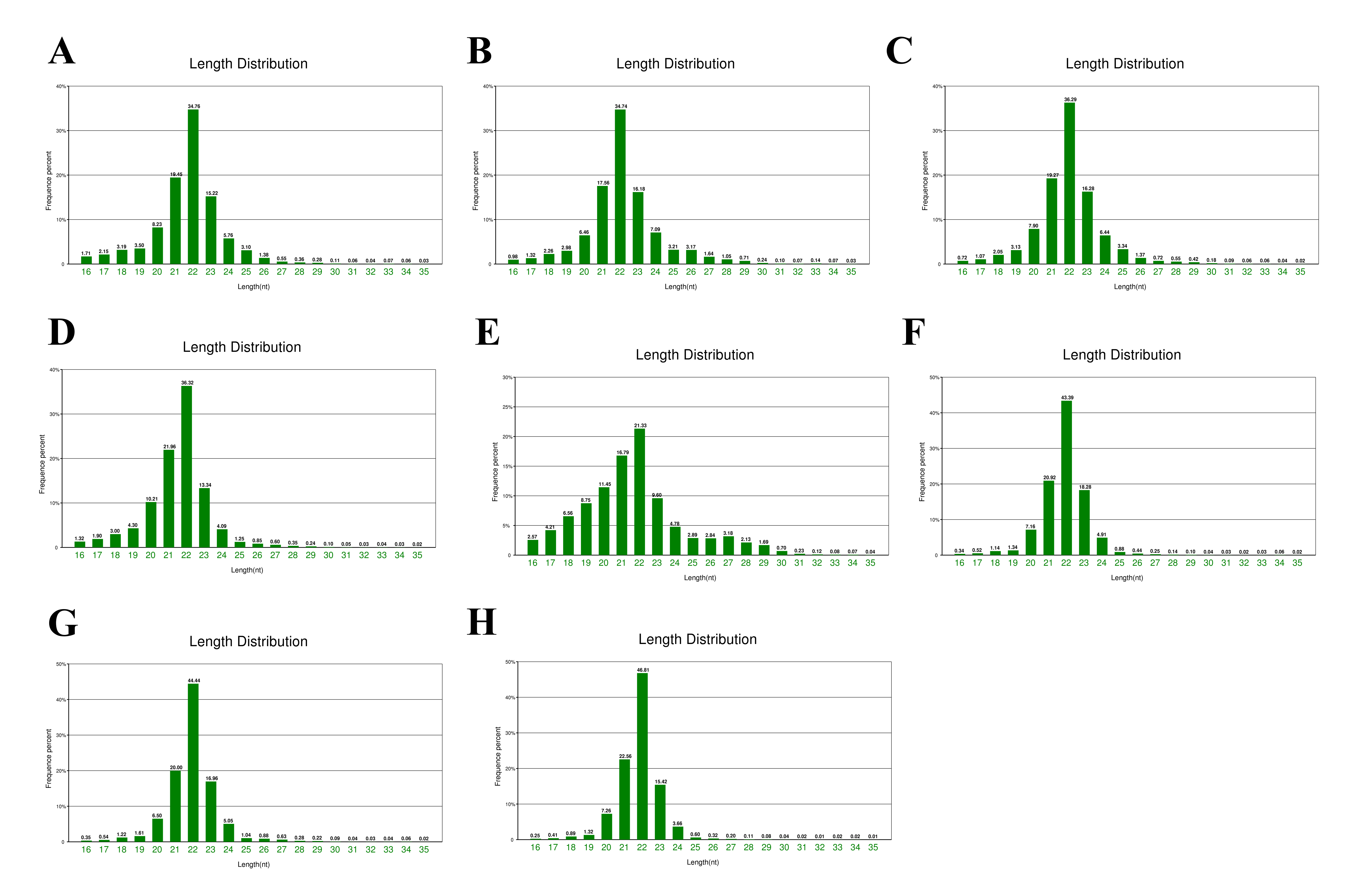

Supplement: Supplementary file 1 [file animals-15-00506-s001.zip › Supplementary Figure S1.png]

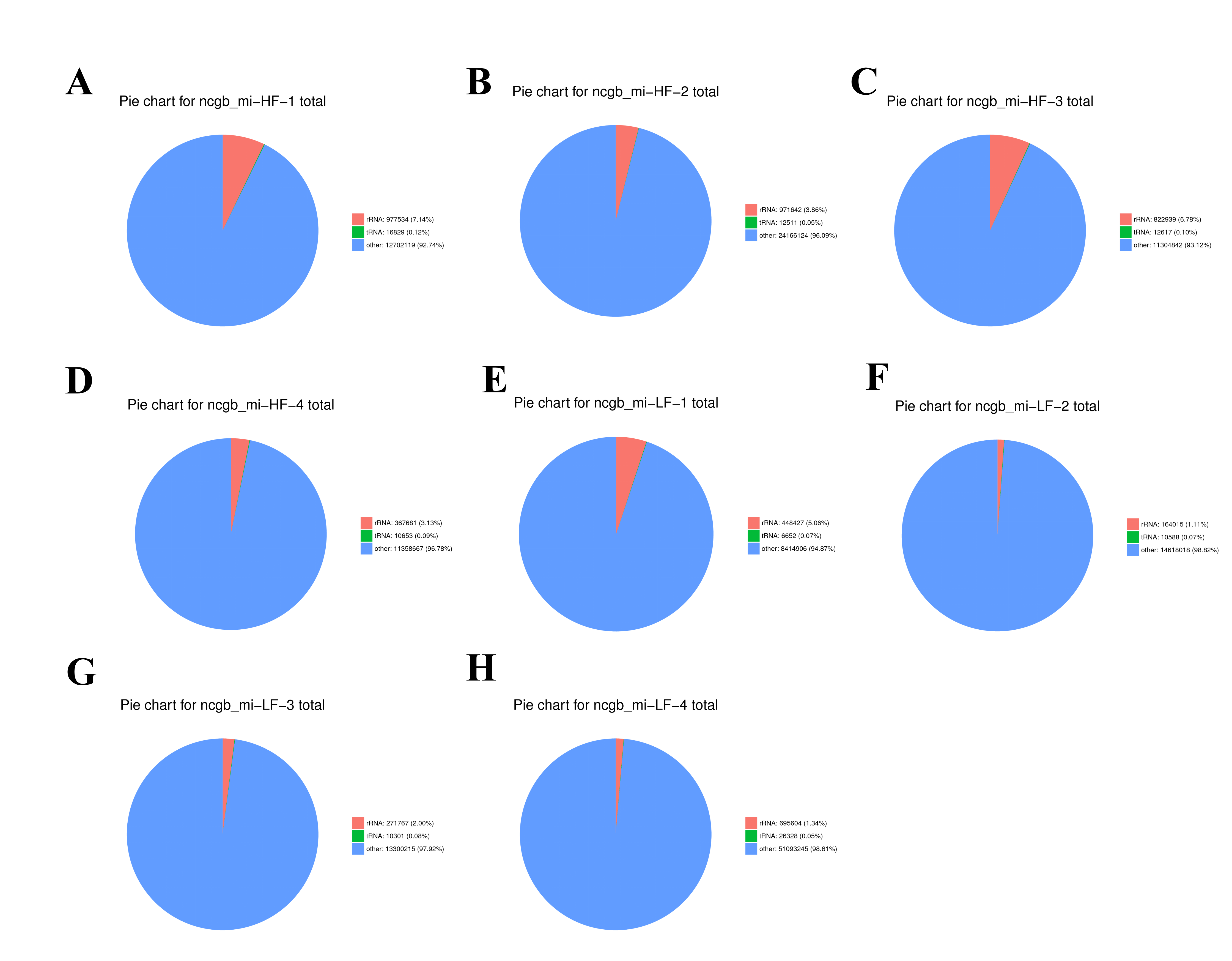

Supplement: Supplementary file 1 [file animals-15-00506-s001.zip › Supplementary Figure S2.png]

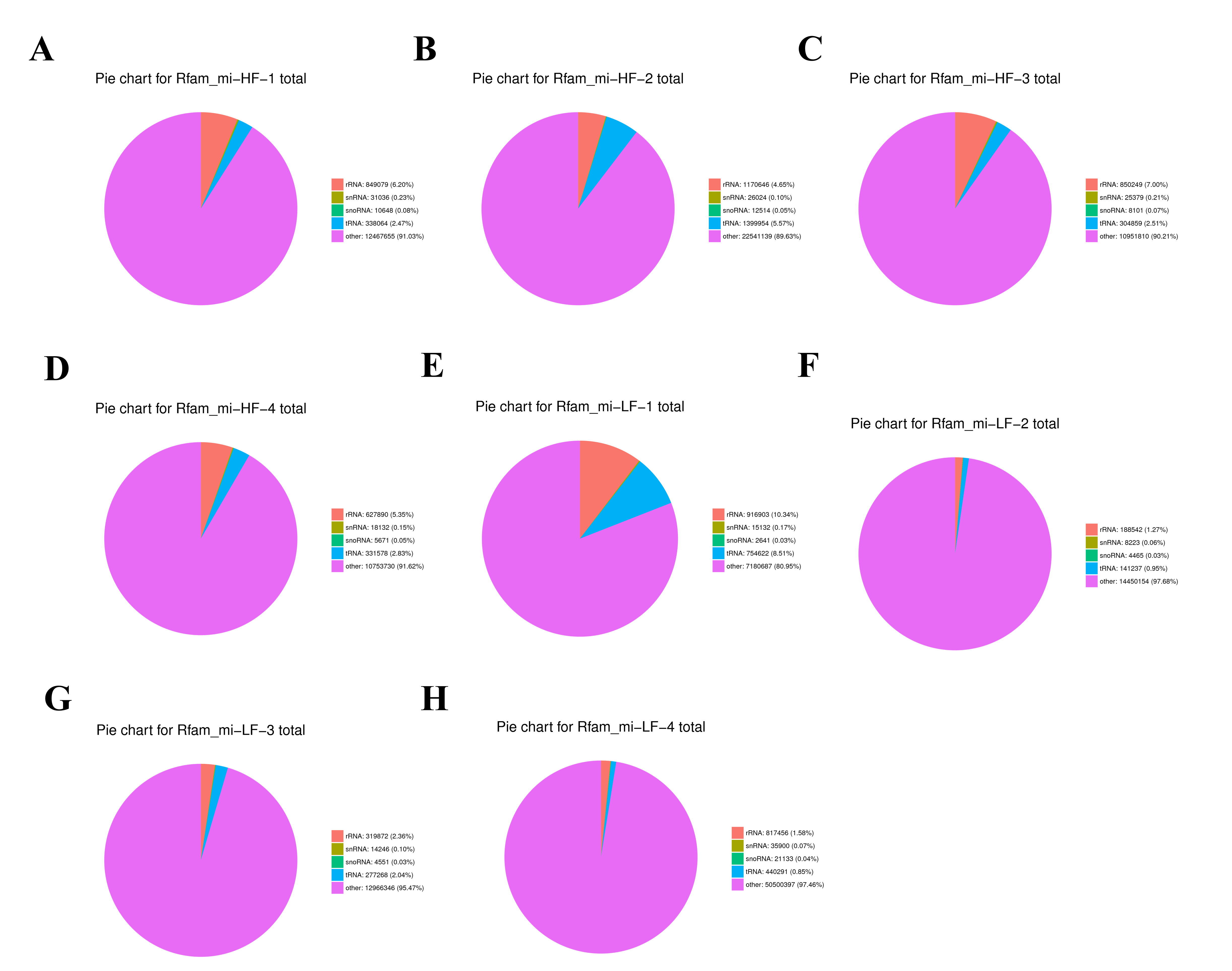

Supplement: Supplementary file 1 [file animals-15-00506-s001.zip › Supplementary Figure S3.png]

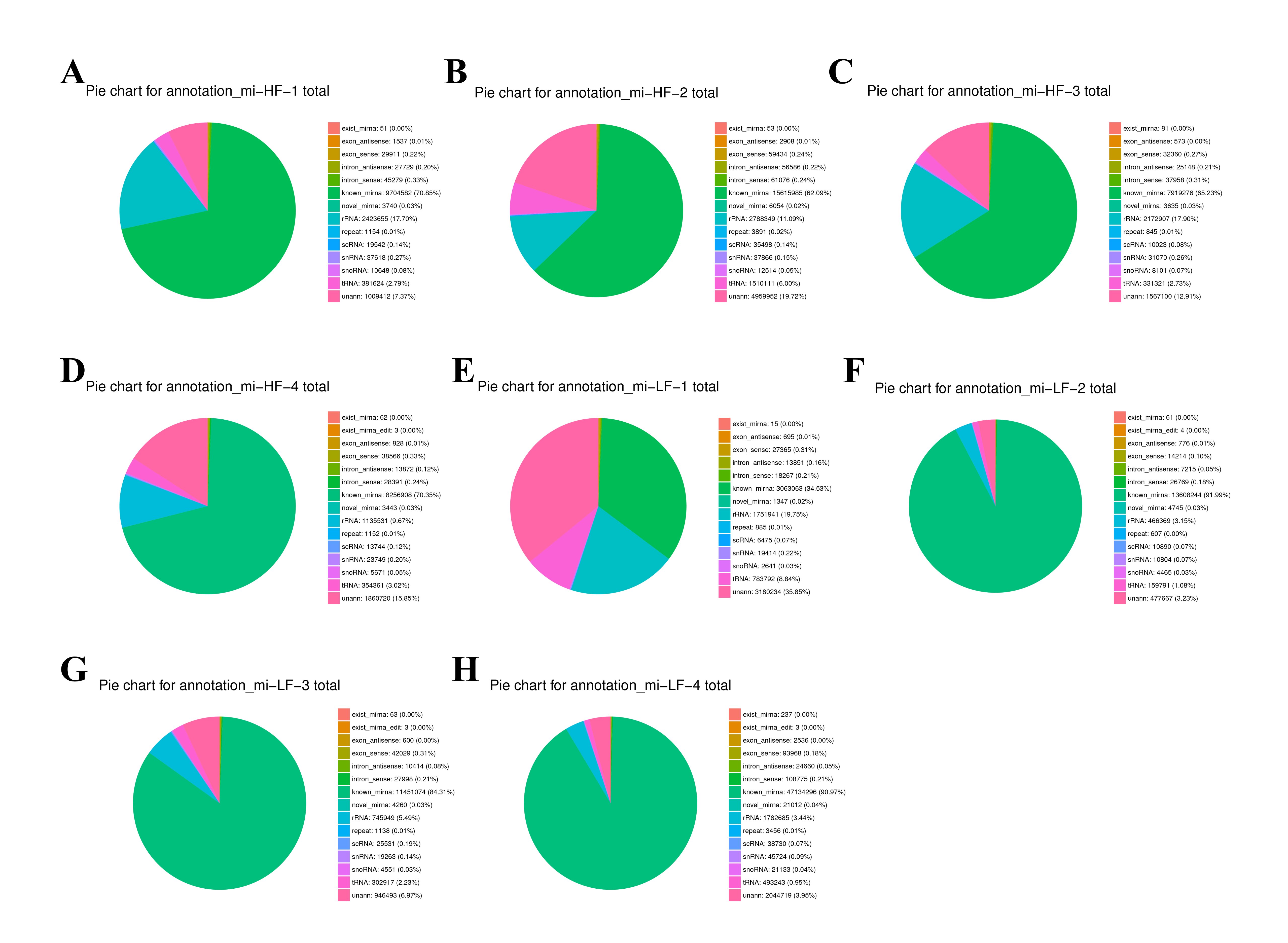

Supplement: Supplementary file 1 [file animals-15-00506-s001.zip › Supplementary Figure S4.png]
